# Editorial: Advances in the research of diabetic retinopathy

**DOI:** 10.3389/fendo.2022.1038056

**Published:** 2022-10-25

**Authors:** Subrata Chakrabarti, Michele Lanza, Khalid Siddiqui

**Affiliations:** ^1^ Western University, London, ON, Canada; ^2^ University of Campania Luigi Vanvitelli, Caserta, Italy; ^3^ Strategic Center for Diabetes Research, College of Medicine, King Saud University, Riyadh, Saudi Arabia

**Keywords:** diabetic retinopathy, pathogenesis, mechanisms, diagnosis, treatment

Diabetes remains a planetary crisis with its prevalence estimated to increase by nearly 50% in the next 25 years (1, 2) One of the main challenges for the diabetic patients is the development of chronic complications, leading to end organ damage. Chronic diabetic complications are a major cause of mortality and morbidity for the people living with diabetes. Retinal damage in diabetics, also known as diabetic retinopathy (DR) is a leading cause of blindness in working-aged adults (3, 4). Although DR begins with asymptomatic hyperglycemic damage to the retinal microvasculature, in particularly endothelial cells, it eventually causes a symphony of abnormalities at various levels, creating cellular dysfunction and damage, ultimately leading to functional and structural changes in the retina that result in vision impairment and blindness (5–7). Current treatment approaches serve as band-aid solutions that address the root of the problem in a very limited perspective (8, 9). To develop a solid preventive and therapeutic approach for DR, a better understanding of this disease is essential.

This Research Topic presents a large number of articles including original research as well as review to improve our understanding of DR. The specific topics represent various levels of complexities. The publications address issues ranging from the molecular level to cellular level to animal levels and finally to DR patients. The articles investigate and discuss specific pathogenetic mechanisms, effects of current treatment modalities and treatment outcomes.

It was also to be noted that this collection also identifies potential upcoming treatment modalities and diagnostic approach using various RNA molecules as well as application of artificial intelligence and machine learning for DR diagnosis and assessment of prognosis.

Among the review topics Zhang et al. compared effectiveness of panretinal photocoagulation alone along with in combination of anti VEGF treatment. Guo et al. discussed uric acid abnormalities, an understudied area in DR. Similarly, Zheng et al. reviewed another relatively unappreciated topic, i.e, relationship of sleep quality with risk of developing DR. Furthermore, Zhang et al. reviewed the effects of calcium dobesylate treatments in patients with non-proliferative DR.

As we are entering in an area of RNA based therapy and diagnosis, two of the presented articles provided valuable insight in this area. Niu et al. reviewed exosomes/microRNAs in the treatment of DR and Kowluru discussed Long Noncoding RNAs and Mitochondrial Homeostasis in the Development of DR. Practical applications of these reviews these reviews were also supported by original research articles. In one such article Biswas et al. described the use of a serum lncRNAs panel to diagnose DR. In the other article Yang et al. characterised small RNAs and microRNAs in the vitreous Humor of proliferative DR. It was also revealed that plasma metabolomic profiling (Sun et al.) growth differentiation factor 15 levels (Niu et al.) may also be effective in the assessment of DR.

Several researchers presented original research describing roles of various additional molecules in DR, including secreted cystine rich acidic protein (Luo et al.), homocysteine ( Luo et al.) and retinal inflammation and macrophage infiltration (Meng et al.).

The articles presented in this Research Topic further showed the impact of glycemic control on photoreceptor Layers and RPE in type 2 Diabetes (Ishibashi et al.). Furthermore, Xie et al. showed assessment of fundus structure using OCT may act as a predictive model for treatment effect for diabetic macular edema. Best corrected visual acuity in macular edema may also be predicted by OCT (Li et al.). Deng et al. proposed the use of hand-held ERG device for DR Screening. In addition, it was shown that artificial intelligence and machine learning may also predict DR in type 2 diabetic patients (Zhao et al.). It was also important to note the data of Kailuan eye study (Yongpeng et al), showing that DR is an independent risk factor for dry AMD.

In conclusion, this Research Topic brings insights and a wealth of knowledge regarding DR, involving its pathogenesis, diagnostic modalities, clinical presentation and treatment. We sincerely feel that these set of articles have set the stage for new knowledge creation and their clinical application in this area in future.

## Author contributions

SC drafted the manuscript. ML and KS reviewed, provided input and approved the content.

## Conflict of interest

The authors declare that the research was conducted in the absence of any commercial or financial relationships that could be construed as a potential conflict of interest.

## Publisher’s note

All claims expressed in this article are solely those of the authors and do not necessarily represent those of their affiliated organizations, or those of the publisher, the editors and the reviewers. Any product that may be evaluated in this article, or claim that may be made by its manufacturer, is not guaranteed or endorsed by the publisher.
